# Evolution of Microstructure, Texture and Mechanical Properties for Multilayered Al Matrix Composites by Accumulative Roll Bonding

**DOI:** 10.3390/ma14195576

**Published:** 2021-09-26

**Authors:** Wen-Jing Wang, Kam-Chuen Yung, An-Dong Tang, Hang-Shan Choy, Zheng Lv

**Affiliations:** 1 School of Materials Science and Engineering, University of Science and Technology Beijing, Beijing 100083, China; tangad1112@163.com; 2 Institute for Advanced Materials and Technology, University of Science and Technology Beijing, Beijing 100083, China; 3 Beijing Laboratory of Metallic Materials and Processing for Modern Transportation, Beijing 100083, China; 4 Department of Industrial and Systems Engineering, The Hong Kong Polytechnic University, Hung Hom, Kowloon, Hong Kong 100077, China; wincokc.yung@polyu.edu.hk (K.-C.Y.); choy.henry@polyu.edu.hk (H.-S.C.); 5 Advanced Electronic Materials Institute, GRIMAT Engineering Institute Co., Ltd., Beijing 101407, China; lvzheng1988@126.com

**Keywords:** accumulative roll bonding, metal matrix composites (MMCs), carbon nanotubes and nanofibers, mechanical properties

## Abstract

Carbon nanotubes (CNTs) reinforced aluminum matrix nanocomposites were fabricated by Accumulative Roll Bonding (ARB). The surface morphologies, mechanical properties, grains texture and orientation of the Al/CNTs nanocomposites were characterized, and the mechanisms and influences of CNTs contents and ARB cycles on the mechanical performance and grain textures of Al/CNTs were investigated and revealed. The strength of the composites rose with increase of the CNTs content, and the ARB cycles showed a 26% improvement when the CNTs content varied from 0 to 1 volume percent (vol.%). The increase in the mass fraction of the carbon nanotubes made the grain distribution in the Al/CNTs nanocomposite samples more diffuse. Besides, the stable texture of the hot rolled crystal grains on the α orientation are constantly turning to {011}< 011> with the mass fraction of the reinforcing phase increased.

## 1. Introduction

Aluminum matrix nanocomposites have attracted considerable interest for structural design due to their excellent mechanical and physical properties. The influence of the reinforcement is clearly present via high strength and high modulus than the sample with no reinforcement [1]. The ceramic nanoparticles in the Al matrix could increase the porosity and decrease the density to fabricate lightweight composites [2]. These excellent properties offer wide opportunities in applications such as aerospace and automotive structural components. During the last decade, various types of nanosized reinforcements have been used in an aluminum matrix to synthesize nanocomposites. Ceramic nanoparticles reduced grain sizes at higher ARB cycles, which caused additional strengthening [3]. A recent study showed the increased tensile strength and microhardness of the Al8011/SiC with only up to ARB three cycles [4]. However, the elongation of the Al/MWCNT-Al_2_O_3_ hybrid composite is decreased by ten cycles of ARB [5].

Carbon nanotubes (CNTs), which have engendered considerable attention as reinforcements in metal matrix composites, present high strength, high elastic modulus as well as high aspect ratio [6,7,8]. For structural applications, Al/CNTs composites have been successfully processed using different techniques, and most investigations have followed the powder metallurgy route, accompanied by the plastic deformation process. For instance, ball milling and spark plasma sintering for consolidating the composites have been applied [9,10]. Researchers have also explored the melt route (such as spraying and liquid infiltration) and the severe plastic deformation route (SPD, such as high-pressure torsion, ECAP, and roll bonding). To prepare composites with high strength and elastic modulus offers a possibility of fabricating materials by using multi-laminates or introducing particles or films with the repeated processes of accumulating and rolling [11]. In theory, full diffusion of particles could always be obtained with sufficient ARB cycles in the composites fabricated. Furthermore, a metal matrix composite fabricated by the ARB process is not necessary to process additional densification treatment. However, to date, few studies have been conducted on the investigation of Al/CNTs composites processed by the ARB process. The mechanism and influence of the ARB process on the microstructure and mechanical properties of Al/CNTs composite are limited and not clear.

The present work aimed at preparing Al/CNTs composites using the ARB process and in revealing the microstructures and mechanical properties of the composites. In addition, the morphologies of CNTs within the Al matrix, and the grain and macrotexture of the Al matrix were analyzed using transmission electron microscopy (TEM), electron back-scattered diffraction (EBSD) and X-ray diffraction (XRD) methods. The mechanical properties of composites were investigated by tensile tests.

## 2. Experimental Section

### 2.1. Materials Preparation

**Figure 1:**
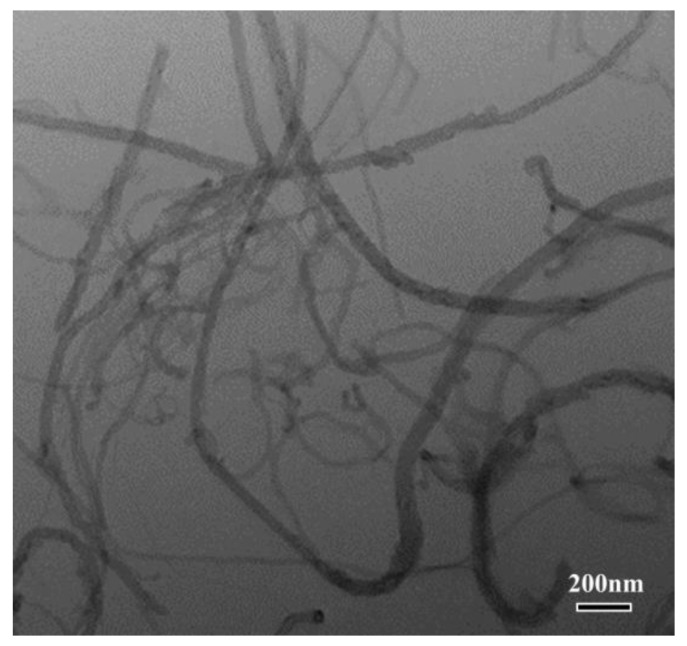
TEM micrograph of MWCNTs used in this work.

Sheets of commercial pure aluminum alloy AA1060 (H24, GB/T 16475-2008) were used as rolled samples, with dimensions of 100 × 50 × 0.6 mm. Multi-walled carbon nanotubes (MWCNTs) with outer diameters of 10–30 nm and a nominal axial length of 10–20 μm were used as reinforcements (Figure 1).

Before ARB, the surfaces of the Al sheets were scratched with a stainless steel brush and then degreased in acetone. CNTs were dispersed on one-side of six raw Al sheets only. In this study, the addition of CNTs was obtained by ultrasonic-assisted liquid phase deposition. This deposition process consisted of two main steps. The first step was to prepare CNTs suspension liquids. Disperbyk-2150 was used in the CNTs suspension to obtain the excellent compatibility, flowing property and the increased content of CNTs [12]. Multipolymer Disperbyk-2150 (BYK Additives Co., Ltd., Shanghai, China) was added to ethanol with a volume percentage of about 40%, and then the CNTs were mixed with the as-prepared solution by ultrasonicated treatment for 30 min. The second step was to disperse CNTs on the surfaces of the Al sheets. The prepared Al sheets were bathed in a suspension liquid filled with CNTs, and then the suspension solution was ultrasonicated again until the ethanol evaporated.

The roll bonding process was performed without lubrication, and the prepared sheets were heated to 673 K for 30 min and then operated a single pass with 50% rolling reduction and speed of 1 m/min. Subsequently, the composite sheet was cut into two halves and stacked for next ARB cycle. The whole ARB process was repeated for up to 12 cycles. The monolithic Al sheet was also prepared using the same process as the contrast. In this study, three compositions were prepared through the ARB process: pure aluminum and Al/CNTs composites with 0.5 and 1.0 vol.% CNTs.

### 2.2. Materials Characterization

JEOL Model JSM-6490 (JEOL Ltd., Tokyo, Japan) and JEM2010 (JEOL Ltd., Tokyo, Japan) TEM was applied for microstructure and grain structure observations and analysis. The EBSD samples were electrolytic polished in a 10% HClO_4_ ethanol solution at 20 V for 10 s. FE-SEM equipped with an HKL EBSD system was used to collect microstructure information. The overall texture was evaluated by X-ray diffraction (XRD, Rigaku SmartLab, Rigaku Corporation, Tokyo, Japan). Incomplete pole figures of {1 1 1} {2 0 0} {2 2 0} were recorded, and the ODFs (Orientation Distribution Functions) were calculated from the three incomplete pole figures using the series expansion method. A servohydraulic universal testing machine (CMT4105, Suns, Shenzhen, China) was used to test various ARB sheets at a nominal strain rate of 1.0 × 10^−3^ s^−1^ at ambient temperature.

## 3. Results and Discussions

### 3.1. Morphology of Al and Al/CNTs Composites

**Figure 2:**
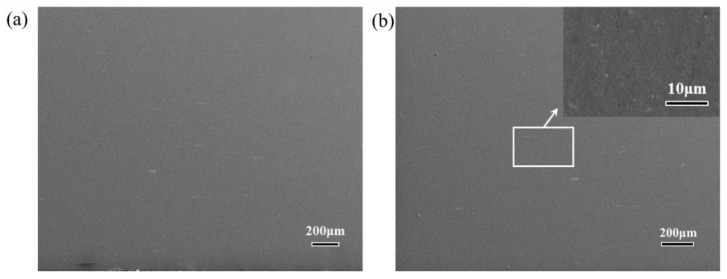
SEM micrographs of (**a**) pure Al and (**b**) Al/1.0 vol.% CNTs processed by 12 ARB.

Improvements in the mechanical properties of composites prepared by the ARB process, especially nanocomposites, depend on effective bonding of the sheets and uniform distribution of the reinforcements. Figure 2 shows the SEM micrographs for the rolling direction (RD) and normal direction (ND) of pure Al and Al/1.0 vol.% CNTs nanocomposites processed over 12 ARB cycles. It can be seen that there were no obvious interfaces in the two compositions after the 12 cycles.

**Figure 3:**
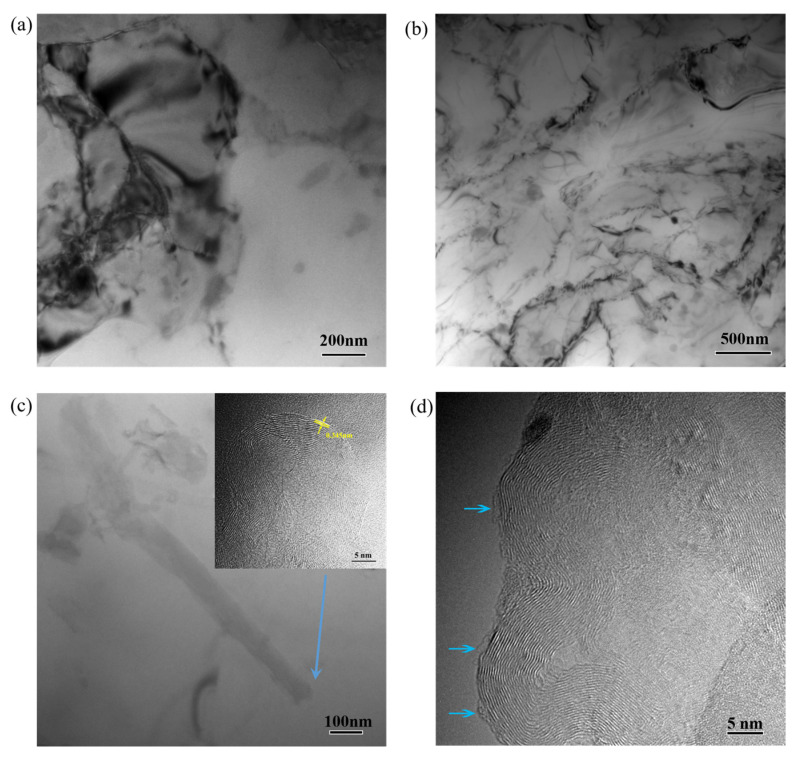
Typical TEM images of Al/1.0 vol.% CNTs nanocomposites processed by 12 ARB cycles. (**a**) Substructures in Al matrix; (**b**) deformed structures in Al matrix; (**c**) individual MWCNT within Al grain; (**d**) MWCNT cluster at grain boundary.

The typical TEM images of Al/1.0 vol.% CNTs nanocomposites processed over 12 ARB cycles are shown in Figure 3. Typical substructures (Figure 3a) and deformed structures (Figure 3b) were found in the composite. For the deformed structures, lots of aggregative dislocations were found to be in the form of dislocation networks. Figure 3c,d shows the CNTs distribution in the composite. The CNTs were found to be singly dispersed in the Al grain (Figure 3c), while some CNT clusters were distributed along the grain boundaries (as shown by the black arrow in Figure 3d). The interlayer spacing of CNTs, calculated by using a Digital Micrograph, was 0.345 nm, consistent with the ideal graphitic interlayer space (0.34 nm). The distribution of CNTs at the grain boundaries exerted an effective pinning on the grain boundaries, contributing to the generation of many dislocations in the composites.

From Figure 3d, the surfaces of the CNTs were covered with an amorphous carbon layer, as shown by the blue arrow. Careful inspection indicated that there were no obvious reaction layers at the interface of CNTs and Al matrix in the composite. Al_4_C_3_ is one kind of the interfacial reaction compounds, and excessive interfacial reaction compounds would be detrimental to the interfacial bonding of the composites [13,14]. Al/CNTs composites prepared by the PM route, and brittle phase Al_4_C_3_ at the interface of the CNTs and Al matrix, caused severe impact damage to the CNTs during the long-time ball milling, thereby the consolidation with high temperature (~870 K) led to the covalent bonding between the Al matrix and the defective CNTs [15,16,17]. In this work, the CNTs during one ARB cycle were only subjected to a single force perpendicular to their axial direction at a relatively lower temperature process (673 K). Consequently, the CNTs were more stable in the Al matrix and did not have some carbide formation.

### 3.2. Mechanical Properties

**Figure 4:**
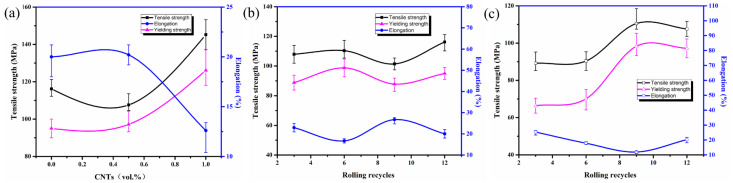
Mechanical properties of Al/CNTs nanocomposites with 0, 0.5 and 1.0 vol.% CNTs after 12 ARB cycles (**a**) and Al with 0 (**b**), 0.5 (**c**) vol.% CNTs rolling different cycles.

**Figure 5:**
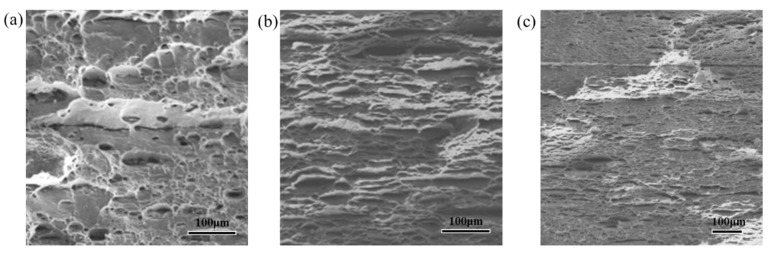
Tensile fracture morphologies of Al/CNTs nanocomposites with 0 (**a**), 0.5 (**b**) and 1.0 (**c**) vol.% CNTs after 12 ARB cycles.

The tensile strength tests results with different CNTs content are shown in Figure 4a. The strength with CNTs content increasing from 0~1.0 vol.% decreased first, then increased sharply at 0.5 vol.% Al/CNTs. This is due to different mechanical properties of the constituent layers [18]. It is evident that the flow properties of Al are different from those of CNTs. During the ARB process, the plastic instabilities such as necking and fracture occur as a result of the difference between the flow properties of the Al and CNTs layers. The necking and fracture of layers are affected by the strain hardening exponents of the constituent layers, strength coefficients of the constituents, and initial thickness ratio of the constituents [19]. The dimples of tensile fracture of pure aluminum laminated sheets are obvious and deep. However, the elongation after fracture decreases to a certain extent with the increased volume fraction of CNTs. In the meanwhile, it is also shown reduced dimples and subdued plastic fracture, as shown in Figure 5.

The tensile strength test results of pure Al and Al with 0.5 vol.% CNTs content after rolling 3, 6, 9 and 12 cycles are shown in Figure 4b,c. The tensile strength and yield strength of Al with 0.5 vol.% CNTs both show an upward trend with increasing cycles, but both the strengths of the intermediate cycle pass show a saltation occasionally. However, the yield strength of pure Al shows a different trend. By increasing ARB cycles, the thickness of the layers decreases, and the interface of the layers gradually tends to a wavy shape. As necking extends, the shear bands are created in the composite after middle ARB cycles. Thus, the presence of plastic instabilities in the fabricated composites at the final ARB cycles decreases their tensile strength. Since CNTs and Al layers are forced together during ARB process, a strain gradient is necessarily generated in their interface to fit the different strains in these layers. By increasing the strain (increasing the number of ARB cycles), geometrically necessary dislocations (GNDs) are created and pile-up near the interfaces of CNTs and aluminum layers. The dislocation pile-up in the interface of the CNTS and Al layers produces a back stress strengthening [20].

### 3.3. Texture Analysis

**Figure 6:**
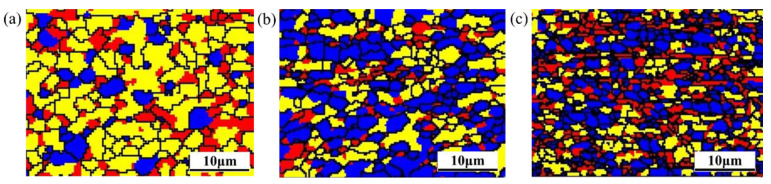
EBSD recrystallization maps of the pure Al (**a**) and Al/CNTs nanocomposites with 0.5 (**b**) and 1.0 (**c**) vol.% CNTs after 12 ARB cycles. The blue, yellow and red grains are denoted as recrystallized, substructured and deformed grains, respectively. RD-ND section with RD parallel to scale bar.

The rolling cycles with a high degree of recrystallization has a significant decrease in strength. On the contrary, the strength of the material with fewer cycles is lower. Additionally, the substructured grain frequency (shown in Figure 6) decreases with the increasing content of CNTs, and the work-hardening effect caused by substructured grains outweighs the texture softening effect. The recrystallized, substructured and deformed frequency of pure Al hot-rolled for 12 cycles was 14%, 61% and 25%, respectively. Meanwhile, those of Al/0.5 vol.% CNTs and Al/1.0 vol.% CNTs hot-rolled 12 cycles was 48%, 38%, 14% and 40%, 29%, 31%, respectively. The strengthening effect caused by the dispersion of CNTs and deformation, and the variation of the deformed grain frequency shown in Figure 6 has a consistent trend with the strength in Figure 4.

The increase of carbon nanotubes content increases the number of grain boundaries in the composites. The number of grain boundaries directly depends on the grain size, so the increase of tensile strength can be explained by the effect of grain boundaries on the plastic resistance of polycrystals. According to the Hall–Petch effect, the strength of polycrystal increases with the increase of carbon nanotubes and grain refinement. As the number of rolling cycles and CNTs content increases, the grains transform from small-angle grain boundaries to large-angle grain boundaries, and this transformation eventually constructs a thin layer structure, which can be seen from the delamination of the grain structure. When the number of rolling cycles is further increased, the thin layer structure is broken and finally transformed into fine equiaxed grains. According to the Hall–Petch formula, the smaller the grain size of the material, the greater the strength of the material. Additionally, the large strain results in the increase inside dislocation density and the defects, and the dislocation slip movement is blocked, which is manifested as an increase in strength.

**Figure 7:**
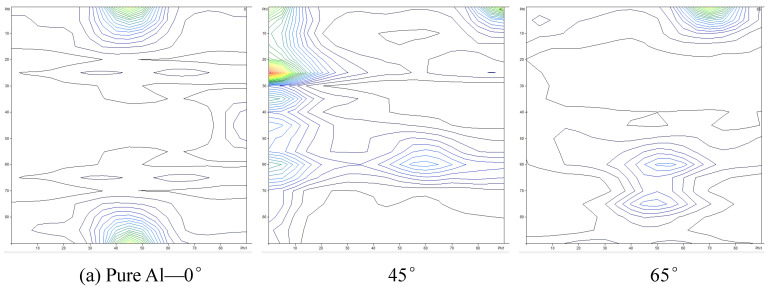

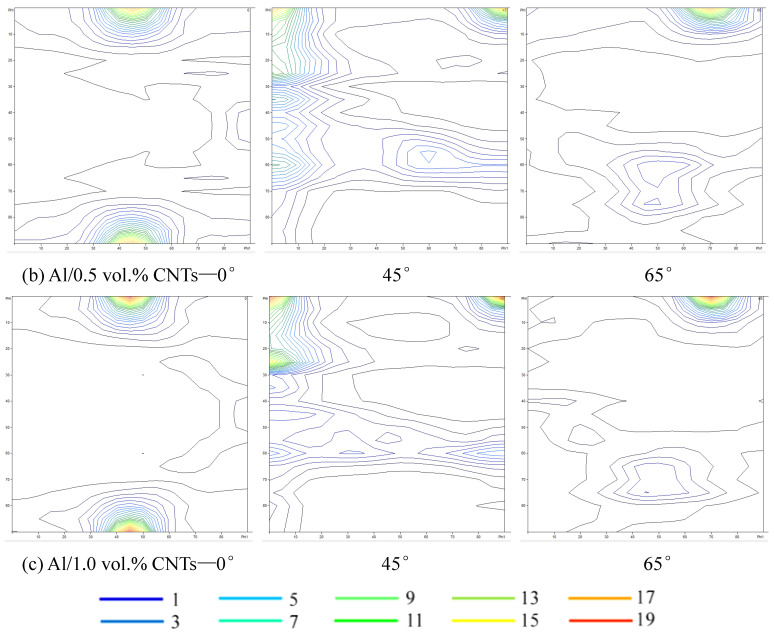
The orientation distribution functions (ODFs) (φ_2_ = 0°, 45°, and 65° sections) for rolling textures of the pure Al (**a**) and Al/CNTs nanocomposites with 0.5 (**b**) and 1.0 (**c**) vol.% CNTs after 12 ARB cycles.

Figure 7 presents the orientation distribution functions (ODFs) at φ_2_ = 0°, 45°, and 65° sections for the pure Al and Al with 0.5, 1.0 vol.% CNTs, respectively. Compared with the typical preferred crystallographic orientations in rolled face-centered-cubic (FCC) metals, the texture of pure Al was nearly randomly distributed, and rotated cube and copper components appeared with relatively low orientation density, as shown in Figure 7a. There was no novel texture type developed in Al with 0.5, 1.0 vol.% CNTs, as shown in Figure 7b,c. However, the texture density was enhanced from 7.5 to 18.9 with the CNTs content increasing.

**Figure 8:**
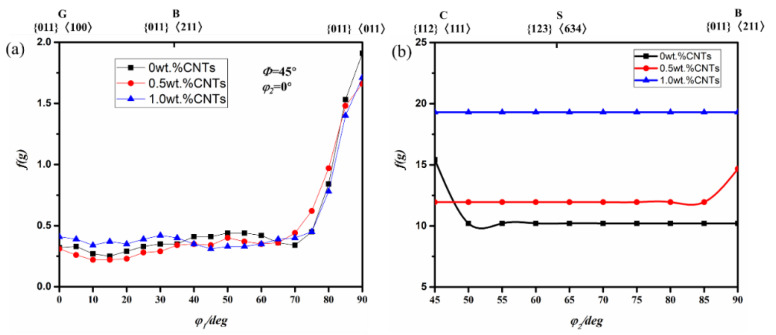
Orientation density f (g) of Al with 0, 0.5, 1.0 vol.% CNTs nanocomposites. (**a**) α orientation line (**b**) β orientation line.

Figure 8 shows the orientation density f(g) of Al with 0.5, 1.0 vol.% CNTs. With the mass fraction of the reinforcing phase increased from 0% to 1%, the grains on the α orientation are constantly turning to {011}<011>, and the mass fractions have little influence on the orientation density. The crystal grains gather toward the β orientation line, but there is a certain degree of deviation from the standard position. The rolling texture is mainly concentrated in the vicinity of the β orientation line and the copper (C) and brass copper (B) orientations. As the mass fraction of the reinforcing phase increases, and the aggregation extent on the β orientation line escalates. As the mass fraction is increased from 0 to 0.5%, the orientation of the grains turn from C orientation to the B orientation constantly. As the mass fraction increases to 1.0%, both the C, S, and B orientations maintained at a higher density.

During the rolling process, the orientation of the crystal grains moves along a certain route to a stable orientation, and the final stable orientation of Al/CNTs is {011}<011> orientation. In the ARB process, the carbon nanotubes are second-phase particles added artificially. As the rolling deformation continues, the second phase particles added by the ARB cycles and the moving dislocations will be blocked by the particles, making the dislocations bend around the particles. This results in the accumulation of dislocations that continue to increase, which produces a reaction force to the source of the dislocation and increases the resistance to bypassing the dislocation, causing the leading dislocation to slip or climb, making the dislocation loop interact and wrap around the particle. The increased dislocation density in the material produces a work hardening effect. Meanwhile, due to the incompatibility between the carbon nanotube particles and the surrounding aluminum matrix particles, a large number of dislocations are generated around the particles. These dislocations can ensure the geometric integrity of the material, but also produce a work hardening effect. Both of these dislocations will hinder the activation of the sliding system.

Although the few second-phase additions make the crystal turning to the hard orientation and stop slipping after the slip system is activated, it will still cause some potential slip system in the harder orientation previously activated. The movement system results in multiple slip occurring. In the ARB pure Al and Al/CNTs, because the strengthening effect of the second phase particles does not increase linearly with the content of the particles, the second phase particles in the face-centered cubic aluminum metal will also affect the activation of the slip system. Moreover, the composition and intensity variation of the rolling texture are affected by the dispersion strengthening effect and multi-slip effect of the second phase particles.

## 4. Conclusions

The mechanical properties of the Al/CNTs nanocomposites are affected by the content of CNTs and the ARB cycles. The increase of the strength of the composite is due to the transformation from the small angle grain boundaries to the large angle grain boundaries because of severe plastic deformation, and the eventual formation of ultra-fine grains and the high density of dislocations by ARB process and the enhancement effect of the carbon nanotubes enhanced phase.

The hot rolling stable texture is {011}<011> after 12 cycles. The increase in the mass fraction of the carbon nanotubes makes the distribution of grains in the sample more diffuse. Under the combined effect of the strengthening of the second phase particles and the multi-slip, the rolling texture strength of the nanocomposite increases with increase in the mass fraction of the phase.

The composition and distribution of the rolling texture can be improved by controlling the quality score of the enhancement item, and then controlling the microstructure and organization of the material, and optimizing the comprehensive performance of the material, according to actual application requirements. The study has laid a theoretical foundation for the development of Al/MWCNTs nanocomposite materials, which is beneficial to the development and promotion of Al/MWCNTs nanocomposites in the aerospace field.

## Data Availability

Not applicable.
